# Gene expression of *INPP5F* as an independent prognostic marker in fludarabine-based therapy of chronic lymphocytic leukemia

**DOI:** 10.1038/bcj.2015.82

**Published:** 2015-10-02

**Authors:** G Palermo, D Maisel, M Barrett, H Smith, G Duchateau-Nguyen, T Nguyen, R-F Yeh, A Dufour, T Robak, D Dornan, M Weisser

**Affiliations:** 1 Roche Pharma Research and Early Development, Innovation Center, Basel, Switzerland; 2 Roche Pharma Research and Early Development, Innovation Center, Penzberg, Germany; 3 Hoffman-La Roche Pharmaceuticals Ltd, Welwyn, UK; 4 Hoffman-La Roche Ltd, Basel, Switzerland; 5 Biostatistics, Genentech, Inc., South San Francisco, CA, USA; 6 Laboratory for Leukemia Diagnostics, Klinikum Grosshadern, Ludwig Maximilians University, Munich, Germany; 7 Department of Haematology, Medical University, Lodz, Poland, South San Francisco, CA, USA; 8 Research Oncology Diagnostics, Genentech, Inc., South San Francisco, CA, USA

## Abstract

Chronic lymphocytic leukemia (CLL) is a heterogeneous disease. Various disease-related and patient-related factors have been shown to influence the course of the disease. The aim of this study was to identify novel biomarkers of significant clinical relevance. Pretreatment CD19-separated lymphocytes (*n*=237; discovery set) and peripheral blood mononuclear cells (*n*=92; validation set) from the REACH trial, a randomized phase III trial in relapsed CLL comparing rituximab plus fludarabine plus cyclophosphamide with fludarabine plus cyclophosphamide alone, underwent gene expression profiling. By using Cox regression survival analysis on the discovery set, we identified *inositol polyphosphate-5-phosphatase F* (*INPP5F*) as a prognostic factor for progression-free survival (*P*<0.001; hazard ratio (HR), 1.63; 95% confidence interval (CI), 1.35–1.98) and overall survival (*P*<0.001; HR, 1.47; 95% CI, 1.18–1.84), regardless of adjusting for known prognostic factors. These findings were confirmed on the validation set, suggesting that *INPP5F* may serve as a novel, easy-to-assess future prognostic biomarker for fludarabine-based therapy in CLL.

## Introduction

Chronic lymphocytic leukemia (CLL) is generally an incurable disease. However, the clinical course of the disease is heterogeneous. Although some patients experience rapid progression and rapid need of antileukemic therapy, others remain stable and are observed for many years. This heterogeneity is also reflected in the response to therapy and in long-term clinical outcomes such as progression-free survival (PFS) and overall survival (OS). Prognostic factors that influence patient outcomes consist of cytogenetic rearrangements such as del(17p) and del(11q), molecular factors such as the *immunoglobulin variable region heavy chain* (*IGVH*) mutational status, p53 mutational status and expression of cell surface markers such as CD38 and ZAP70.^1^ Clinical factors such as age, stage of disease and lymphocyte doubling time have also been shown to have prognostic relevance.^2^ For patients in need of therapy, chemoimmunotherapy such as the combination of fludarabine and cyclophosphamide with the anti-CD20 monoclonal antibody rituximab (R-FC) has been demonstrated to significantly prolong PFS and OS compared with the combination of fludarabine and cyclophosphamide alone (FC) in untreated (first-line) patients^3^ as well as to improve PFS in previously treated (second-line) patients.^4^ The objective of this study was to evaluate prognostic and/or predictive biomarkers for rituximab-based therapy from the REACH trial. Recently, we reported that higher expression levels of *PTK2* mRNA were associated with improved PFS in patients treated with R-FC compared with FC alone, whereas no significant differences were observed between the two treatment arms in patients with lower *PTK2* expression levels,^5^ demonstrating predictive significance for rituximab-based chemoimmunotherapy. Here, we demonstrate the prognostic relevance of *inositol polyphosphate-5-phosphatase F* (*INPP5F*) gene expression, a novel, easily assessable biomarker in relapsed CLL.

## Materials and methods

REACH (NCT0090051) was an open-label randomized (1:1) phase III trial in relapsed CLL comparing FC with R-FC. The primary end point of the study was to demonstrate prolonged PFS of R-FC compared with FC alone. The study protocol was approved by institutional review boards at participating centers and all patients gave written informed consent. Details on trial design and eligibility criteria and clinical outcome have been described previously.^4^ Patients were selected based on the availability of sufficient RNA and had to provide written informed consent to participate in the additional studies.

### Gene expression profiling

Pretreatment samples for molecular profiling analysis were available from 237 of 552 (55%) patients enrolled in the REACH trial, selected by the availability of adequate quality and quantity of RNA and on whether the patients gave their informed consent to further molecular analysis. Peripheral blood mononuclear cells (PBMCs) were obtained by Ficoll density gradient centrifugation. CLL samples were positively enriched by magnetic cell sorting using CD19 microbeads and MACS columns (Miltenyi Biotec GmbH, Bergisch Gladbach, Germany) and resuspended in RLT buffer (Qiagen, Crawley, West Sussex, UK). In addition, as a validation set, PBMC samples (*n*=92) were processed into RLT buffer without CD19 enrichment. RNA from both sets was subsequently isolated using Ambion miRVANA total RNA extraction kit (Life Technologies, Carlsbad, CA, USA). Gene expression profiling was performed using Affymetrix U133 Plus 2.0 full transcriptome oligonucleotide arrays according to the manufacture-recommended protocol (Affymetrix, Santa Clara, CA, USA).

### Expression-level computations

The probe intensities of mRNA HG-U133 Plus 2.0 array were background-subtracted, quantile-normalized and summarized using the RMA method^6^ within each sample type (CD19^+^ and PBMCs). Data were further normalized by an empirical Bayes approach (Combat)^7^ to remove potential batch effects due to data acquisition. All data used in this work were log_2_ transformed. Probe sets with very low expression (log_2_-transformed expression levels <4.0 in >95% of samples) were excluded and a total of 26 453 probe sets (out of 54 675) were considered for further analysis.

### Statistical analysis

To test for a possible selection bias on the baseline patient characteristics for each mRNA subset (CD19+ and PBMCs) as compared with the overall REACH population, the χ^2^ test or Wilcoxon–Mann–Whitney test was used respectively for binary and continuous variables. Similar statistical tests were used to check whether the baseline patient characteristics were balanced between FC and R-FC within each mRNA subset. The predictive and/or prognostic utility of the mRNA was assessed using the following approach. First, a log-likelihood ratio test (with two degrees of freedom (2 d.f.)) was used to compare the Cox proportional hazards model of PFS including treatment, mRNA (as continuous) and treatment–mRNA interaction as covariates (full model) against the same model but with the interaction term being absent (reduced model). Use of a join test (2 d.f.) allowed simultaneous searching for both prognostic and predictive markers, significantly increasing the power to detect predictive markers compared with a 1-d.f. treatment–mRNA pure interaction test (based on simulations; data not shown). The *q*-values (false discovery rate)^8^ were calculated from raw log-likelihood ratio test *P-*values to account for multiple hypothesis testing and only mRNAs with a *q*-value <1% were considered significant. At this stage, the full model was considered again and the treatment–mRNA interaction term was tested to decide whether a significant marker was predictive or prognostic. In addition, to assess whether candidate mRNAs provide predictive or prognostic information independently of known prognostic factors, the mRNA was also evaluated in the context of an expanded, multivariate Cox proportional hazards model that included a parsimonious set of known prognostic factors chosen (using a forward stepwise selection procedure) among the following variables: age; Binet stage; *IGVH* mutational status; chromosome 17p, 11q and 13q deletions; trisomy 12; β2-microgloblulin; lymphocytic count; and Eastern Cooperative Oncology Group (ECOG) performance status.

All analyses were conducted using R (http://www.r-project.org).

## Results

### Patient characteristics

Gene expression profiling data were available from 237 CD19-enriched samples (CD19^+^ discovery set: 115 within the FC arm; 122 within the R-FC arm) and 92 PBMC samples that were not enriched for CD19 (PBMC validation set: 46 within the FC arm; 46 within the R-FC arm) of 552 patients enrolled in the REACH trial. The baseline patient demographics and tumor characteristics are shown in Table 1 for the overall REACH population, as well as for the CD19^+^ and PBMC subpopulations with available mRNA (mRNA study population). Summaries from the table suggest that the mRNA study population was representative of the REACH overall study population^4^ (formal statistical tests for each variable in Table 1 did not show any statistical difference between either CD19+ or PBMC mRNA subsets as compared with the overall population) and that the two treatment arms were well balanced with respect to related risk factors, such as age, stage, high-risk cytogenetics and *IGVH* mutational status (again there were no statistically significant differences between FC and R-FC arm within each mRNA subset). In addition, in the mRNA study population, the treatment benefit with respect to PFS (CD19^+^: hazard ratio (HR), 0.69; 95% confidence interval (CI), 0.47–0.99; *P*=0.046; PBMCs: HR, 0.73; 95% CI, 0.42–1.27; *P*=0.26) was comparable with that in the overall population (HR, 0.64; 95% CI, 0.5–0.81; *P*<0.001) as a result of a multivariate Cox regression model adjusting for age, Binet stage, *IGVH* mutational status, ECOG performance status and del(17p). The median follow-up time for PFS for the CD19^+^ and PBMC subpopulations was 23 and 33 months, respectively. The median follow-up time for OS for the CD19^+^ and PBMC subpopulations was 51 and 57 months, respectively.

### *INPP5F* expression and PFS

**Figure 1:**
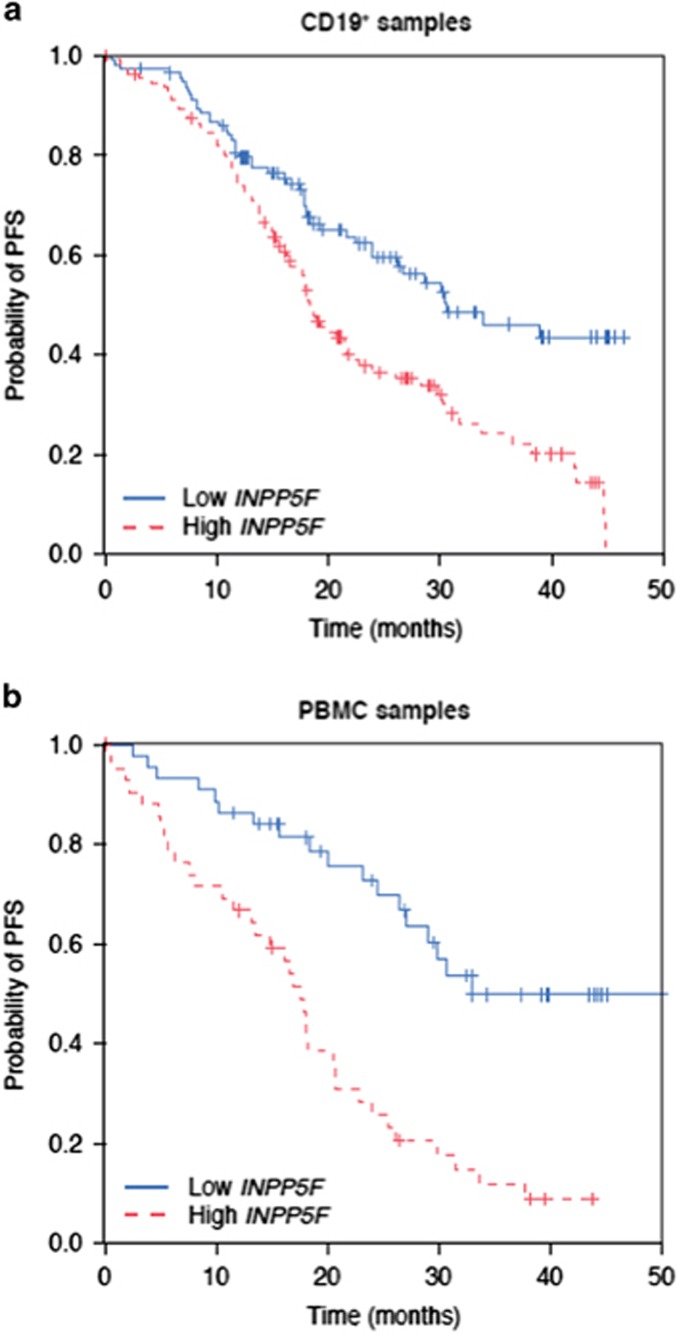
The mRNA isolated from: (**a**) CD19^+^-separated samples and (**b**) PBMCs. Kaplan–Meier curves of PFS stratified by *INPP5F* expression levels (red: high *INPP5F* expression, above the median; blue: low *INPP5F* expression, below the median).

By using the statistical approach described in the Statistical analysis section, applied to the discovery set only (CD19^+^ samples), *INPP5F* expression was identified as a prognostic factor for PFS, regardless of adjusting for treatment, age, Binet stage, *IGVH* mutational status, del(17p) and ECOG performance status (mRNA term *P*<0.001; HR, 1.63; 95% CI, 1.35–1.98 without adjustment; mRNA term *P*<0.001; HR, 1.48; 95% CI, 1.20–1.83 with adjustment),that were selected by a forward-stepwise selection procedure among a larger set of prognostic factors (see Statistical Analysis section). The treatment–mRNA interaction term was not significant in either case (*P*=0.31/0.24 without/with adjustment), suggesting that *INPP5F* is a prognostic rather than predictive marker. When patients were dichotomized into low and high *INPP5F* expression (based on the median expression level of *INPP5F*), lower *INPP5F* expression was associated with improved PFS (median PFS, 30.6 months) compared with higher *INPP5F* expression (median PFS, 18.5 months, Figure 1a).

The prognostic value of *INPP5F* expression on PFS was confirmed in the validation set (PBMC samples) with the mRNA term *P*<0.001 in a Cox regression model regardless of adjusting for age, Binet stage, IGVH mutational status, del(17p) and ECOG performance status (HR, 1.73; 95% CI, 1.33–2.24 without adjustment; HR, 1.83; 95% CI, 1.3–2.6 with adjustment). Again, the treatment–mRNA interaction term was not significant (*P*=0.9/0.9 without/with adjustment). The Kaplan–Meier curves for patients grouped into low and high *INPP5F* expression based on the median expression level are shown in Figure 1b (median PFS, 33 vs 17.6 months, respectively).

In addition to *INPP5F*, the only other genes that demonstrated prognostic relevance for both PFS and OS with respect to expression levels after adjustment of prognostic markers were *PDE8A* (PFS: discovery set HR, 0.63; 95% CI, 0.47–0.84; *P*<0.001; validation set HR, 0.54; 95% CI, 0.35–0.84; *P*=0.0058; OS: discovery set HR, 0.63; 95% CI, 0.45–0.89; *P*=0.008; validation set HR, 0.51; 95% CI, 0.29–0.91; *P*=0.021) and to a lesser extent *MZB-1* (PFS: discovery set HR, 1.46; 95% CI, 1.18–1.81; *P*<0.001, validation set HR, 1.27; 95% CI, 0.97–1.66; *P*=0.085; OS: discovery set HR, 1.38; 95% CI, 1.07–1.79; *P*=0.014, validation set HR, 1.32; 95% CI, 0.92–1.89; *P*=0.14). The prognostic relevance of expression levels of *PDE8A* and *MZB-1* in CLL has been reported previously.^9, 10^

### *INPP5F* expression and OS

**Figure 2:**
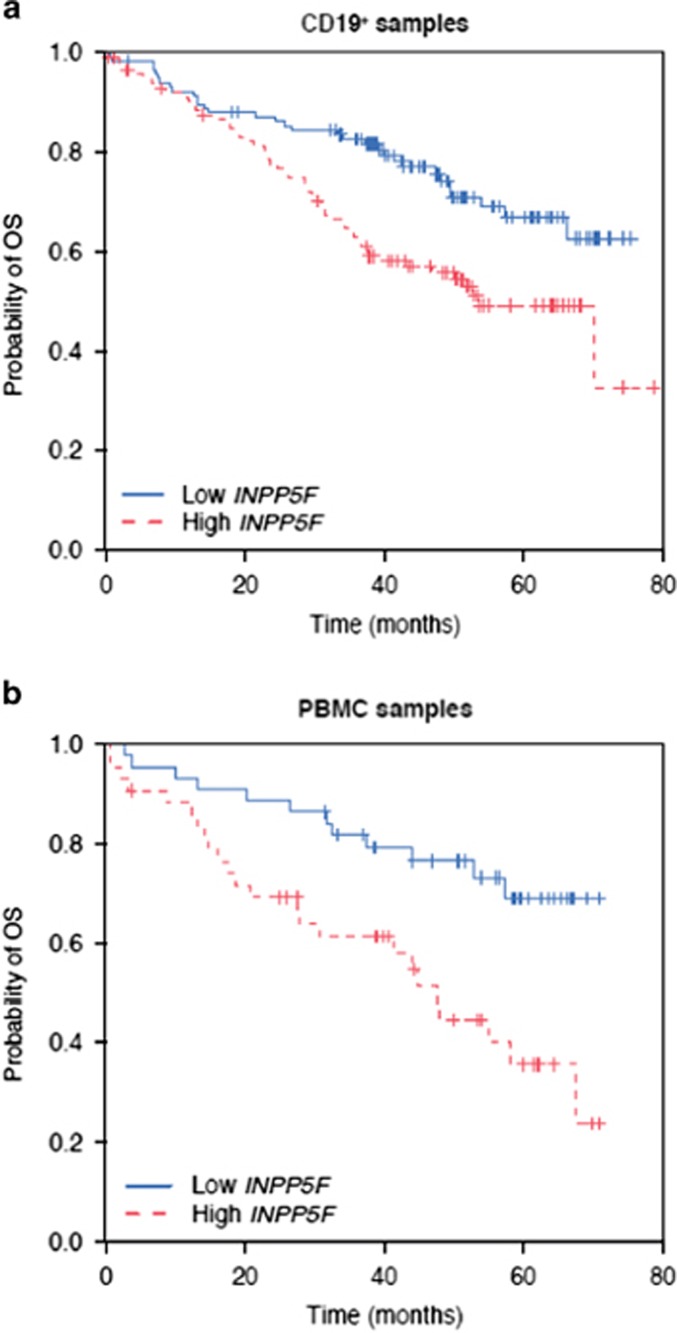
The mRNA isolated from: (**a**) CD19^+^-separated samples and (**b**) PBMCs. Kaplan–Meier curves of OS stratified by *INPP5F* expression levels (red: high *INPP5F* expression, above the median; blue: low *INPP5F* expression, below the median).

In addition, a Cox regression analysis was used to assess the prognostic influence of *INPP5F* expression on OS. In the discovery set (CD19^+^), *INPP5F* expression was significantly associated with OS in a univariate model (*P*<0.001; HR, 1.47; 95% CI, 1.18–1.84) as well as in a multivariate model (*P*=0.013; HR, 1.36; 95% CI, 1.07–1.73) adjusted for treatment, age, *IGVH* mutational status, del(17p), β-microglobulin and ECOG performance status that were selected by a forward-stepwise selection procedure among a larger set of prognostic factors (see Statistical Analysis section). Again, these findings were confirmed in the validation set (PBMCs) with *P*<0.001 (HR, 1.82; 95% CI, 1.32–2.51) in univariate analysis and *P*=0.011 (HR, 1.75; 95% CI, 1.14–2.7) in multivariate analysis. The treatment–mRNA interaction term was not significant in either case (*P*=0.78/0.5 and *P*=0.86/0.98, respectively, for CD19^+^ and PBMC without/with adjustment). The Kaplan–Meier curves of OS for patients grouped into low and high *INPP5F* expression are provided in Figures 2a and b, respectively, for the CD19^+^ and PBMC data set.

**Figure 3:**
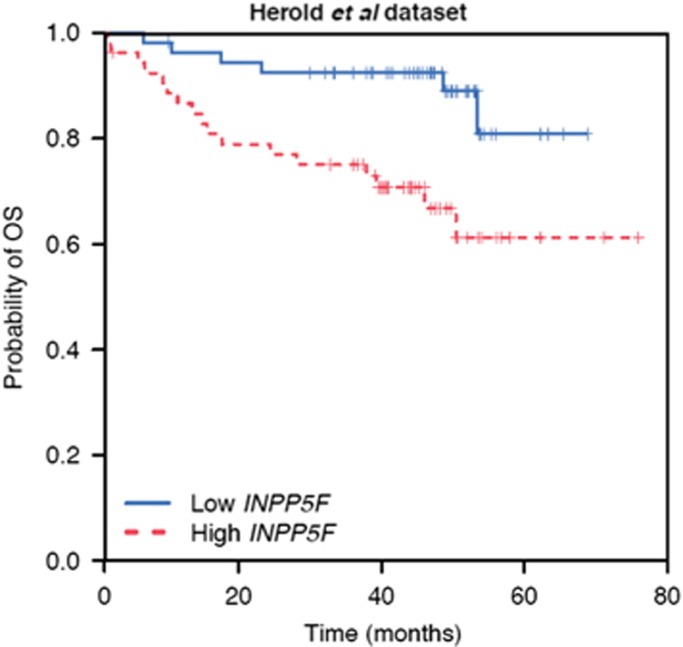
Data set from Herold *et al.*^9^ Kaplan–Meier curves of OS stratified by *INPP5F* expression levels (red: high *INPP5F* expression, above the median; blue: low *INPP5F* expression, below the median).

In addition, *INPP5F* expression measured by Affymetrix HG-U133 Plus 2.0 microarray from a set of 107 patients with newly diagnosed CLL^9^ available in the Gene Expression Omnibus database (http://www.ncbi.nlm.nih.gov/geo/; experiment-ID=E-GEOD-22762) was analyzed for OS. In this additional, independent data set, a significant correlation between OS and *INPP5F* (*P*=0.006; HR, 2.0; 95% CI, 1.2–3.3) was again observed in a univariate Cox regression analysis. *INPP5F* remained significant (*P*=0.02; HR, 1.7; 95% CI, 1.1–2.7) after adjusting for del(17p) and trisomy 12 (a stepwise variable selection did exclude del(11q) and del(13q) from the model; no other predictors were made publicly available). The Kaplan–Meier curves of OS for patients grouped into low and high *INPP5F* expression are provided in Figure 3.

### Correlation of *INPP5F* expression to *BCL-2*, *IKKβ* (*IKKb*) and *IKBα* (*IKBa*)

In the discovery set (CD19^+^), there was a significant correlation of *INPP5F* expression to the expression level of *BCL-2* (*r*=0.4; *P*<0.001). A trend toward statistical significance was observed when *INPP5F* and *BCL-2* expression were correlated in the validation set (PBMCs: *r*=0.20; *P*=0.06). *IKKb* gene expression was found to be significantly correlated to *INPP5F* gene expression in the discovery set (CD19^+^: *r*=0.45; *P*<0.001) as well as in the validation set (PBMCs: *r*=0.32; *P*=0.0015). There was an inverse correlation of *INPP5F* gene expression to *IKBa* gene expression in both the discovery set (CD19^+^: *r*=–0.26; *P*<0.001) and the validation set (PBMCs: *r*=–0.29; *P*<0.001). The expression of *INPP5F* was correlated to the expression of other genes involved in the nuclear factor (NF)-κB signaling cascade (see Supplementary Figures 1a–l and Supplementary Table 1).

## Discussion

*INPP5F* is one of the several polyphosphoinositide phosphatases whose role has been partially elucidated.^11^ *INPP5F* degrades PIP2 (phosphatidylinositol 4,5-bisphosphonate) and PIP3 (phosphatidylinositol 3,4,5-trisphosphonate) regulating AKT/phosphatidylinositol 3-kinase (PI3K) signaling.^12, 13, 14, 15^ *INPP5F* is predicted to reduce PIP3 levels and subsequently reduce AKT/PI3K signaling, thereby attenuating expression of antiapoptotic molecules and leading to increased chemotherapy sensitivity. Because of this, our data showing that lower *INPP5F* levels are associated with improved outcome and higher *INPP5F* levels with poorer outcome in CLL may seem contradictory. However, it has been demonstrated that *INPP5F* acts as a regulator of PIP3 levels. Stimulation of the AKT/PI3K pathway with insulin-like growth factor in *INPP5F* knockout mice led to a substantial increase in *PIP3* levels compared with *INPP5F* wild-type mice,^16^ suggesting a feedback loop of *INPP5F* when the ATK/PI3K pathway is stimulated. In addition, unlike PTEN, which degrades PIP3 to PI(4,5)P2, *INPP5F* degrades PIP3 to PI(3,4)P2, which can function as a second messenger. Furthermore, PI(3,4)P2 activity has been shown to correlate with AKT activity.^15^

In CLL, the NF-κB signaling pathway has been shown to be constitutively active,^17^ resulting in downstream overexpression of antiapoptotic BCL-2 family members.^18, 19^ BCL-2 and BCL-2 family members have been shown to be upregulated in CLL and are also shown to be associated with an unfavorable prognosis^20, 21, 22, 23, 24, 25, 26, 27^ and the connection between BCL-2 and NF-κB has been reported for various hematological malignancies including CLL.^17, 28^

Rituximab has been shown to exert its antitumor activity *in vitro* by attenuating constitutively active AKT and subsequent modulation expression of the antiapoptotic proteins of the BCL-2 family.^29, 30^ Moreover, this rituximab-mediated downregulation of active AKT resulted in sensitization to chemotherapeutics. Low levels of *BCLxl* have been shown to be associated with sensitivity to chemotherapy and CD20-targeted therapy.^29^ In addition, increased levels of BCL-2 family protein have been associated with poorer response to fludarabine and a shorter time to first treatment interval.^23^ We also detected significant correlation of *INPP5F* expression with gene expression of *BCL-2* (*r*=0.4; *P*<0.001) and a significant correlation of *INPP5F* to *IKKb* expression (*r*=0.45; *P*<0.001), whereas for the expression levels of *IKBa*, a significant inverse correlation to the expression of *INPP5F* was observed (*r*=–0.26; *P*<0.001). Interestingly, the *INPP5F* expression level correlated significantly with the expression of *IKKb/IKBKB*, an activator of NF-κB, and was inversely correlated to expression of *IKBa*, an inhibitor of NF-κB,^31, 32^ suggesting an association between high expression of *INPP5F* and the activated NF-κB pathway and an agonistic AKT/PI3K function. However, none of the latter genes demonstrated significant prognostic relevance in the present study.

To our knowledge, this is the first study describing *INPP5F* expression level as a novel prognostic biomarker for PFS as well as OS in a large set of patients treated within a randomized trial. Its prognostic relevance was found in a set of CD19-enriched PBMC samples, and subsequently confirmed in a validation set of unselected PBMCs. The robustness of gene expression profiling for biomarker research in peripheral blood has been demonstrated in multiple previous reports, showing a significant correlation of expression levels between array-based gene expression analysis and reverse transcription-PCR, such as the MAQC Consortium^33^ and also previous publication of outcomes with this same set of samples.^5, 34^ Along these lines, Herold *et al.*^9, 10^ showed significant correlation of *MZB-1* expression in CLL samples when comparing array-based expression profiling with reverse transcription-PCR. In addition, *MZB-1* showed significant correlation to the expression of *INPP5F*, a result that could be confirmed in our discovery set (*r*=0.44, *P*<0.001; Supplementary Materials) and validation set (*r*=0.36, *P*<0.001; Supplementary Materials), further emphasizing the robustness of the reported array data. Further evidence of the prognostic relevance of *INPP5F* levels stems from a subset of untreated (first-line) CLL patients, in whom high expression levels of *INPP5F* mRNA were associated with resistance to R-FC and FC therapy,^35^ as well as a set of previously treated and untreated CLL patients^9^ showing that *INPP5F* expression levels were an adverse prognostic factor for OS. Further research is warranted to understand the biological function of *INPP5F* on survival signaling. *INPP5F* may be a novel, easily assessable biomarker in CLL, because its prognostic significance of *INPP5F* expression remained detectable in the set of unselected PBMCs, and this may support the clinical feasibility of this biomarker as the analytical workflow may not require separation of B cells as for ZAP70 or laborious assessment of the *IGVH* mutational status. To determine the significance of *INPP5F* for prognostic and therapeutic decision making, *INPP5F* cutoff levels need to be defined, and the prognostic relevance of *INPP5F* expression needs to be analyzed in the context of other therapies used for treatment of CLL such as bendamustine.^36, 37^ In addition, our data may also support the strategy of combining NF-κB and/or BCL-2 inhibitors to standard therapy in CLL.

## Figures and Tables

**Table 1:** Patient characteristics

*Characteristic*	*All patients*			*Patients with CD19*^*+*^*-separated mRNA data*			*Patients with PBMC-isolated mRNA*		
	*Total*	*R-FC*	*FC*	*Total*	*R-FC*	*FC*	*Total*	*R-FC*	*FC*
*Sex,* n *(%)*	(*n*=552)	(*n*=276)	(*n*=276)	(*n*=237)	(*n*=122)	(*n*=115)	(*n*=92)	(*n*=46)	(*n*=46)
Female	184 (33)	89 (32)	95 (34)	81 (34)	38 (31)	43 (37)	34 (37)	16 (35)	18 (39)
Male	368 (67)	187 (68)	181 (66)	156 (66)	84 (69)	72 (63)	58 (63)	30 (65)	28 (60)
*Age, years*	(*n*=552)	(*n*=276)	(*n*=276)	(*n*=237)	(*n*=122)	(*n*=115)	(*n*=92)	(*n*=46)	(*n*=46)
Mean (s.d.)	62 (9)	62 (9)	61 (9)	62 (9)	62 (9)	62 (8)	61 (8)	62 (8)	60 (9)
Median (range)	63 (35–83)	63 (35–83)	62 (35–81)	63 (35–83)	63 (35–83)	63 (37–80)	62 (39–81)	63 (39–77)	58 (44–81)
*Race,* n*, (%)*	(*n*=552)	(*n*=276)	(*n*=276)	(*n*=237)	(*n*=122)	(*n*=115)	(*n*=92)	(*n*=46)	(*n*=46)
Caucasian	544 (99)	271 (98)	273 (99)	233 (98)	121 (99)	112 (97)	91 (99)	45 (98)	46 (100)
*Binet stage,* n *(%)*	(*n*=552)	(*n*=276)	(*n*=276)	(*n*=237)	(*n*=122)	(*n*=115)	(*n*=92)	(*n*=46)	(*n*=46)
A	55 (10)	24 (9)	31 (11)	26 (11)	14 (11.5)	12 (10)	8 (9)	4 (9)	4 (9)
B	326 (59)	166 (60)	160 (58)	145 (61)	73 (60)	72 (63)	54 (59)	26 (56.5)	28 (61)
C	171 (31)	86 (31)	85 (31)	66 (28)	35 (29)	31 (27)	30 (33)	16 (35)	14 (30)
*IGVH mutational status,* n *(%)*	(*n*=520)	(*n*=258)	(*n*=262)	(*n*=235)	(*n*=120)	(*n*=115)	(*n*=88)	(*n*=42)	(*n*=46)
Mutated	192 (37)	100 (39)	92 (35)	91 (38)	51 (42)	40 (35)	34 (39)	16 (38)	18 (39)
Unmutated	328 (63)	158 (61)	170 (65)	144 (62)	69 (58)	75 (65)	54 (61)	26 (62)	28 (61)
*Del(17p),* n *(%)*	(*n*=532)	(*n*=269)	(*n*=263)	(*n*=235)	(*n*=121)	(*n*=114)	(*n*=91)	(*n*=46)	(*n*=45)
No	490 (92)	251 (93)	239 (91)	216 (92)	113 (93)	103 (90)	84 (92)	43 (93.5)	41 (91)
Yes	42 (8)	18 (7)	24 (9)	19 (8)	8 (7)	11 (10)	7 (8)	3 (6.5)	4 (9)

Abbreviations: FC, fludarabine and cyclophosphamide; *IGVH*, *immunoglobulin variable region heavy chain*; PBMC, peripheral blood mononuclear cell; R-FC, rituximab plus fludarabine and cyclophosphamide.
