# Supplementary material for: Gene expression of INPP5F as an independent prognostic marker in fludarabine-based therapy of chronic lymphocytic leukemia
Source: Blood Cancer J. 2015 Oct 2;5(10):e353–. doi: 10.1038/bcj.2015.82 (PMC4635191; doi:10.1038/bcj.2015.82)
Supplement: Supplementary Table 1 [file bcj201582x1.docx]

**Supplementary Table 1**

Correlation of gene expression to *INPP5F* expression

|  | **CD19+** | | **PBMC** | |
| --- | --- | --- | --- | --- |
|  | **cor (r)** | ***P* value** | **cor (r)** | ***P* value** |
| **BCL2** | 0.4 | < 0.001 | 0.2 | 0.06 |
| **IKBKB** | 0.45 | < 0.001 | 0.32 | 0.0015 |
| **NFKBIA** | -0.26 | < 0.001 | -0.29 | 0.005 |
| **NFKB1** | -0.47 | < 0.001 | -0.5 | < 0.001 |
| **NFKB2** | -0.42 | < 0.001 | -0.39 | < 0.001 |
| **IKBKE** | 0.42 | < 0.001 | 0.3 | 0.003 |
| **MZB-1** | 0.44 | < 0.001 | 0.36 | < 0.001 |
| **PDE8A** | -0.63 | <0.001 | -0.67 | <0.001 |
