# Supplementary material for: Gene expression of INPP5F as an independent prognostic marker in fludarabine-based therapy of chronic lymphocytic leukemia
Source: Blood Cancer J. 2015 Oct 2;5(10):e353–. doi: 10.1038/bcj.2015.82 (PMC4635191; doi:10.1038/bcj.2015.82)

**Supplementary Figure 1a**

Correlation of gene expression of *INPP5F* to *BCL-2* (CD19+ set)


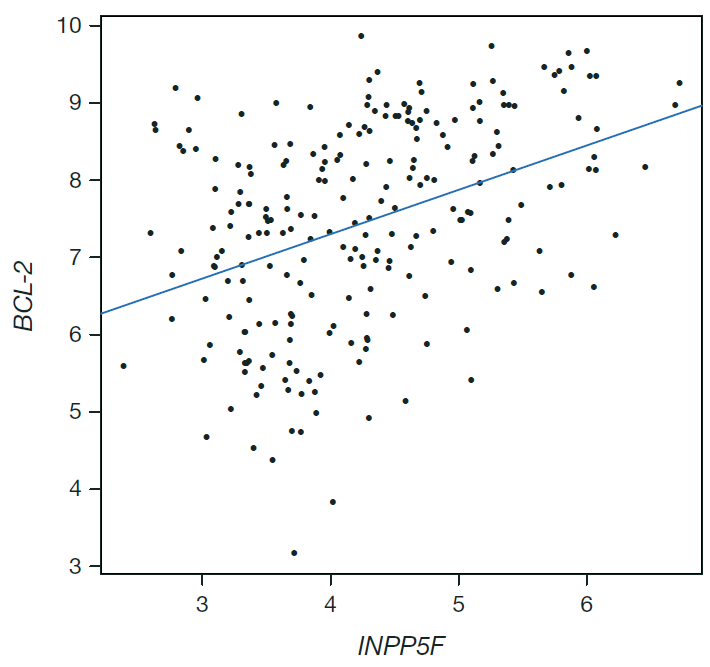


**Supplementary Figure 1b**

Correlation of gene expression of *INPP5F* to *BCL-2* (PBMC set)


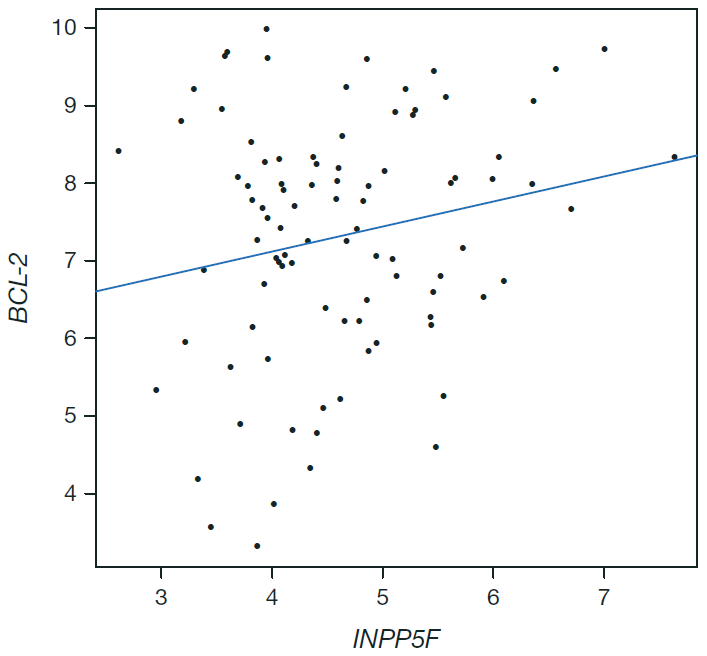


**Supplementary Figure 1c**

Correlation of gene expression of *INPP5F* to *IKBKB* (CD19+ set)


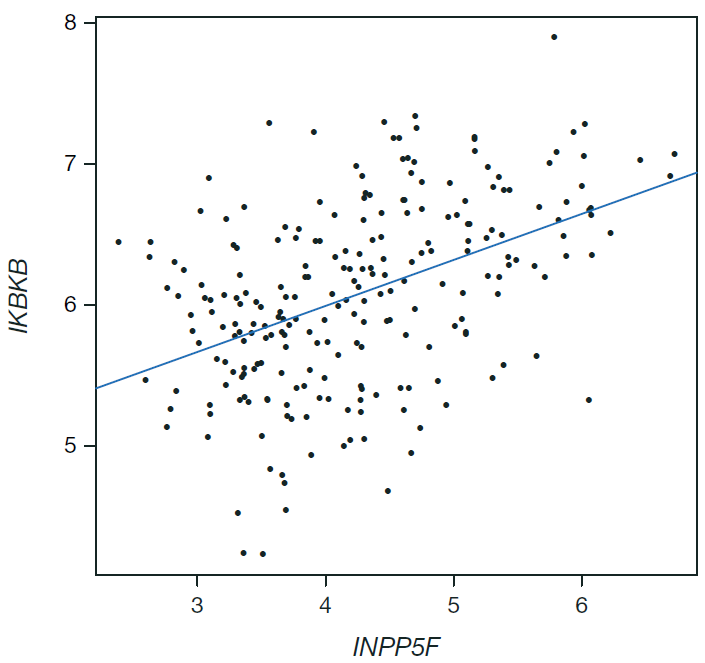


**Supplementary Figure 1d**

Correlation of gene expression of *INPP5F* to *IKBKB* (PBMC set)


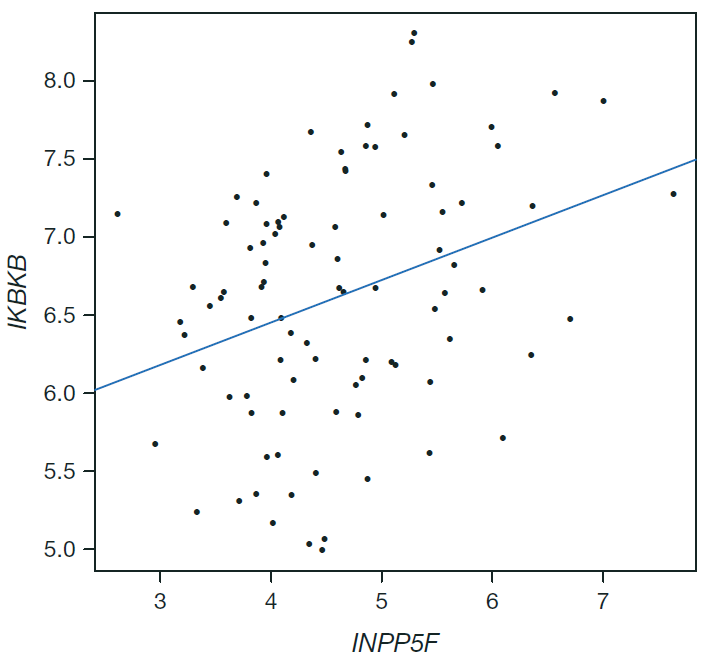


**Supplementary Figure 1e**

Correlation of gene expression of *INPP5F* to *NFKBIA* (CD19+ set)


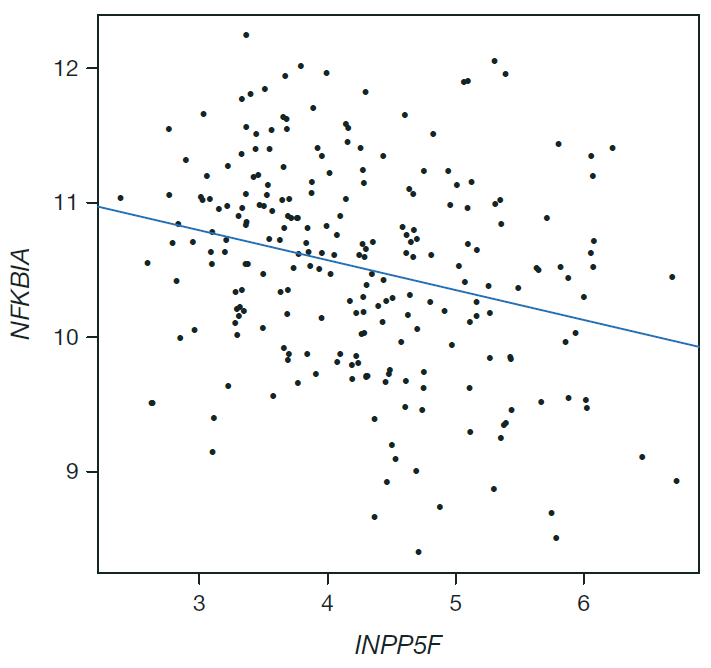


**Supplementary Figure 1f**

Correlation of gene expression of *INPP5F* to *NFKBIA* (PBMC set)


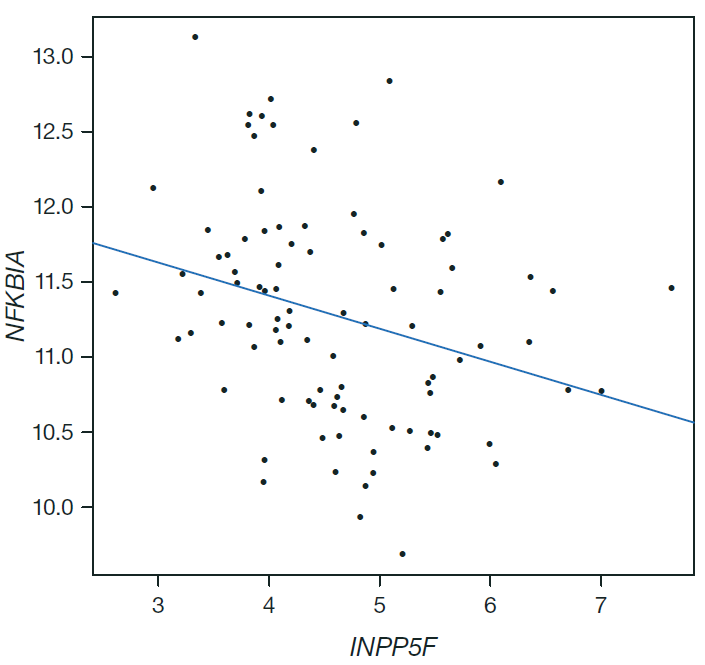


**Supplementary Figure 1g**

Correlation of gene expression of *INPP5F* to *NFKB1* (CD19+ set)


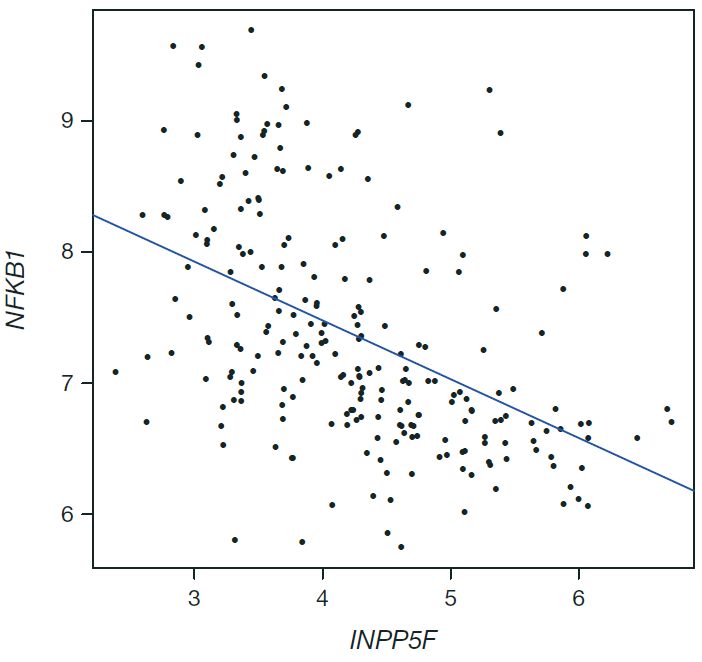


**Supplementary Figure 1h**

Correlation of gene expression of *INPP5F* to *NFKB1* (PBMC set)


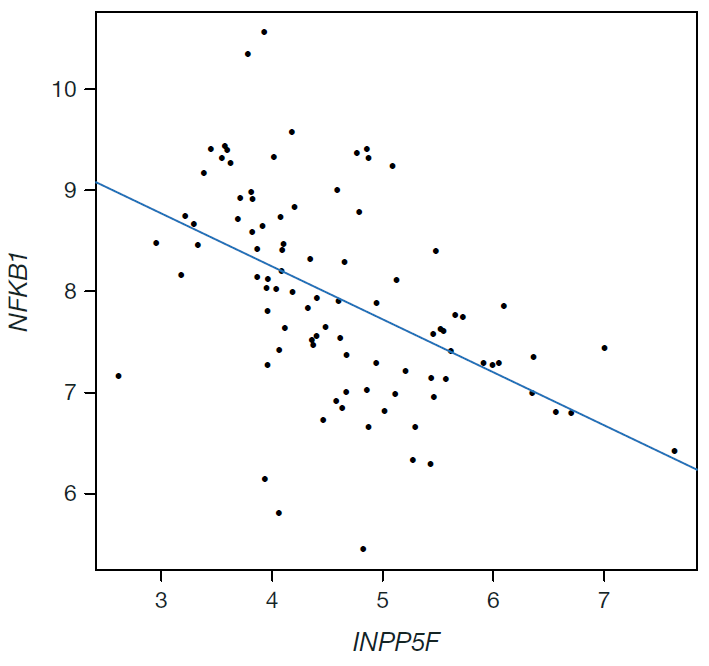


**Supplementary Figure 1i**

Correlation of gene expression of *INPP5F* to *NFKB2* (CD19+ set)


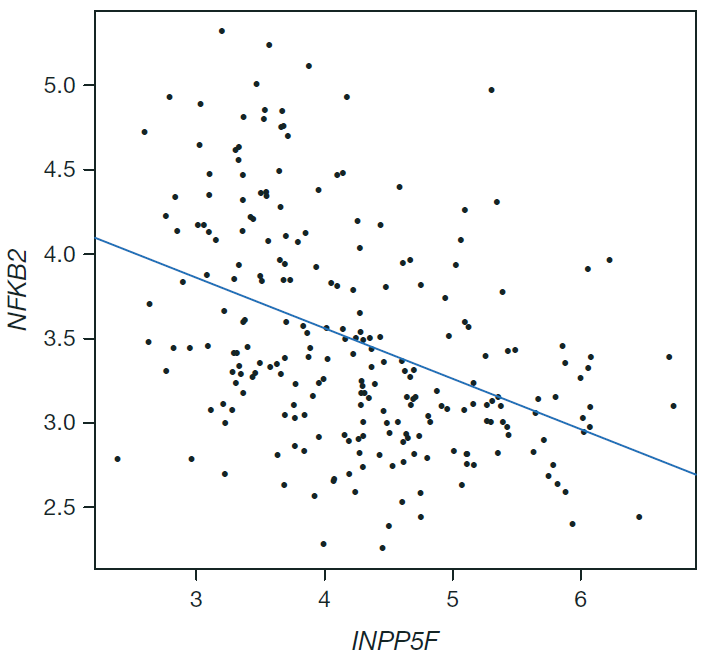


**Supplementary Figure 1j**

Correlation of gene expression of *INPP5F* to *NFKB2* (PBMC set)


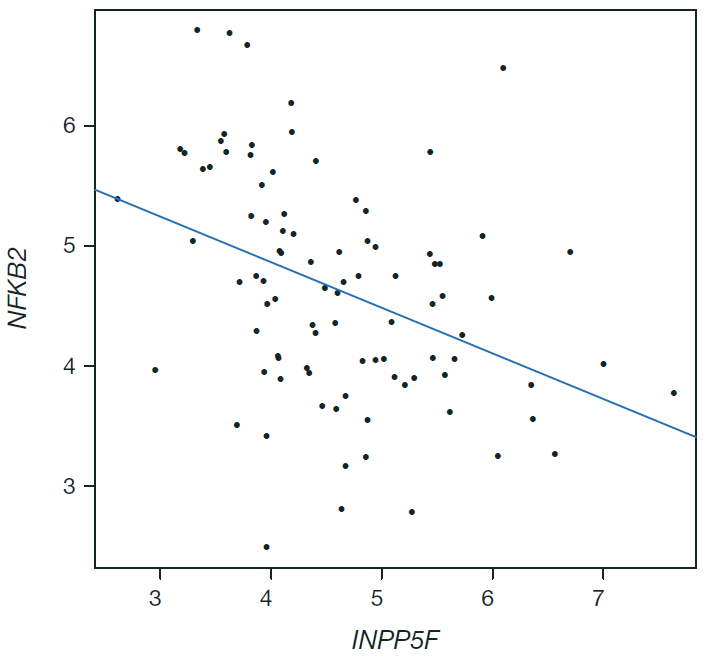


**Supplementary Figure 1k**

Correlation of gene expression of *INPP5F* to *IKBKE* (CD19+ set)


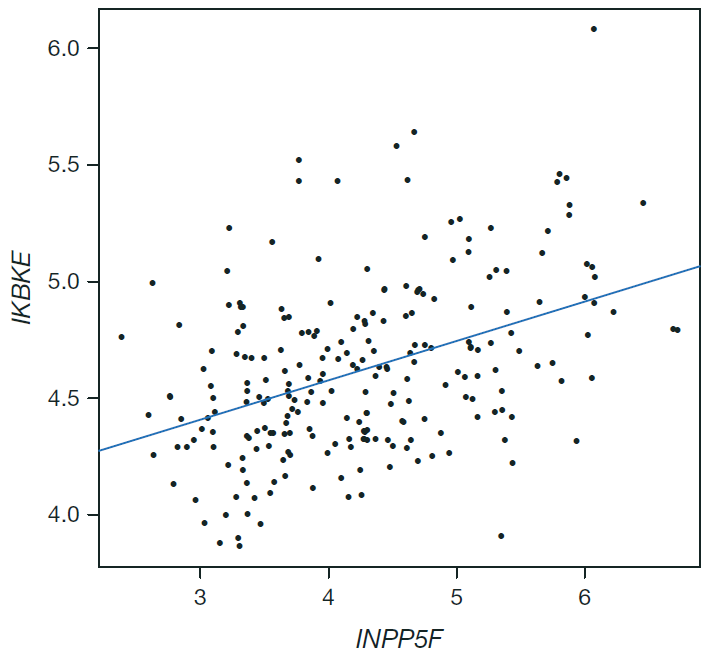


**Supplementary Figure 1l**

Correlation of gene expression of *INPP5F* to *IKBKE* (PBMC set)


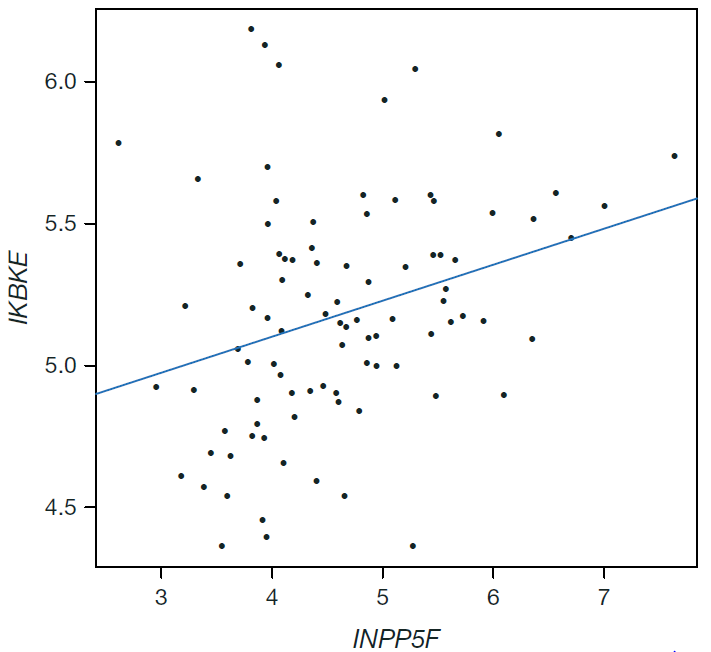

Supplement: Supplementary Figure 1 [file bcj201582x2.docx]
